# Obstetric risk in pregnancy interacts with hair cortisone levels to reduce gestational length

**DOI:** 10.3389/fgwh.2022.878538

**Published:** 2022-07-22

**Authors:** Joseph Musana, Craig R. Cohen, Miriam Kuppermann, Roy Gerona, Anthony Wanyoro, David Aguilar, Nicole Santos, Marleen Temmerman, Sandra J. Weiss

**Affiliations:** ^1^Department of Obstetrics and Gynaecology, Aga Khan University Hospital, Nairobi, Kenya; ^2^Department of Obstetrics, Gynecology and Reproductive Sciences, University of California, San Francisco, San Francisco, CA, United States; ^3^Department of Obstetrics and Gynaecology, Kenyatta University, Nairobi, Kenya; ^4^Clinical Toxicology and Environmental Biomonitoring Lab, University of California, San Francisco, San Francisco, CA, United States; ^5^Institute for Global Health Sciences, University of California, San Francisco, San Francisco, CA, United States; ^6^Department of Community Health Systems, University of California, San Francisco, San Francisco, CA, United States

**Keywords:** perceived stress, cortisol, cortisone, gestational length, obstetric medical risk, fetal sex

## Abstract

**Background:**

Maternal psychological stress has been linked to preterm birth. However, the differential contribution of psychological stress versus stress hormones is not clear. Studies focus primarily on perceived stress and cortisol, with few assessing its inter-convertible hormone cortisone. Furthermore, little is known about the potential moderating roles of obstetric risk and fetal sex in the relationship between maternal stress and gestational length. This gap in knowledge is particularly evident for rural women who typically experience chronic multiple stressors during pregnancy. We explored the relationship of hormonal and psychological stress to gestational length and the effects of obstetric risks and fetal sex on this relationship among Kenyan pregnant women.

**Methods:**

The sample included 130 women recruited between 22 to 28 weeks gestation. They completed a clinical and sociodemographic questionnaire together with the Perceived Stress Scale and provided a hair sample for cortisol and cortisone assay. Women underwent an ultrasound to assess weeks of gestation. At delivery, their pregnancy-related health problems were identified using information extracted from medical records to compile each woman's number of pregnancy risks on the Obstetric Medical Risk Index (OMRI).

**Results:**

Perceived stress and hair cortisol were not significant predictors of gestational length. However, a greater number of obstetric risks on the OMRI was associated with shorter gestational length. This effect was further explained by the interaction between obstetric risk and hair cortisone (*B* = 0.709, *p* = 0.02). Hair cortisone levels of mothers who had a shorter gestation were significantly higher in mothers with 2 or more risks on the OMRI but not among mothers with only one or no risks (*t* = 2.39, *p* = 0.02). Fetal sex had no relationship to gestational length and also had no moderating effect on the relationship between any stress-related metric and gestational length.

**Conclusion:**

Cortisone levels may increase in anticipation of shorter gestation as a compensatory response to increased obstetric risk. Elevated cortisone may be a more sensitive marker of risk for early delivery than cortisol or psychological stress, with salience for both the male and female fetus.

## Introduction

Preterm birth (birth occurring before 37 weeks) is a significant global cause of neonatal morbidity and mortality, with socioeconomic implications and loss of human productivity (1). Research suggests that maternal stress is associated with preterm birth (2, 3). Trends worldwide have shown increased prevalence of preterm birth both in high and low-income countries (4). It is possible that multiple chronic life stressors among pregnant women may contribute to this observed high prevalence in preterm birth and other adverse birth outcomes such as low birth weight and fetal growth restriction (5–9). However, little is known about the mechanisms by which stress influences adverse birth outcomes especially preterm birth (PTB) in this population.

### Psychological distress

Studies in high income countries have shown an association between adverse birth outcomes and various self-reported measures of psychological distress, with mixed results regarding preterm birth (10–23). The largest study, a prospective cohort of 5,511 women conducted in the Netherlands between 2001–2003, found that perceived stress (PS) was associated with infants being born small-for-gestational-age but not with preterm birth (24). However, three other studies in the United States of America with large samples (5,337, 2,533, and 911 women) reported significant associations between PS and PTB, especially in women of low socioeconomic status and coming from disadvantaged minority groups such as those with African American and Hispanic race/ethnicity (25–27). Two studies with a cumulative sample size of 1,550 women indicated that changes in PS rather than levels at a single time point predicted PTB (15, 25).

### Physiologic measures of stress

Because self-report measures of stress may have recall, cultural interpretation, or stigma-related bias, physiologic measures of stress can be important adjuncts in understanding effects of stress. They not only provide objective measurement, but they assess unique components of the stress response beyond self-report. The Hypothalamic-Pituitary-Adrenal (HPA) axis is a major physiological system responsible for coordinating and integrating responses to stress (28). Cortisol and cortisone are the primary effector hormones of the HPA axis (29). Thus, these hormones are a key index of HPA axis activity and ideal physiologic markers to assess. Cortisone is converted from cortisol by the placenta to protect the fetus from excess cortisol. Musana et al. (30) discuss the associations between hair cortisol and perceived stress in pregnant women, citing evidence for both positive and negative associations. Additionally, Hoffman et al. reported hair cortisol to correlate with PS in predicting preterm birth (31). Research also suggests that a larger cortisol awakening response in late pregnancy is associated with shorter gestational length (32). The cortisol/cortisone ratio has been linked to birth weight and cortisone but not cortisol has been associated with risk of pre-eclampsia (33–35). However, there are no documented studies in Sub-Saharan Africa that have examined cortisol and cortisone as measures of the stress response during pregnancy and their association to gestational length. Studies in high income countries are also scarce and with conflicting results (24, 25).

### Obstetric risk

Maternal obstetric risk is a predictor of birth outcomes and may interact with other predictors such as maternal stress and health behaviors to influence preterm birth (36, 37). Obstetric risks can include problems associated with both past pregnancies and the current pregnancy. For example, a history of gestational diabetes, previous stillbirth, placenta previa, or pre-eclampsia as well as current pregnancy-related conditions such as anemia, genitourinary infection, vaginal bleeding, and cervical insufficiency have been associated with premature delivery (38–41). In addition, the cumulative number of such obstetric risks has been linked to preterm birth (42, 43). While the direct influence of these risks on preterm birth has been documented, our understanding of their moderating role in the relationship between pregnancy stress and preterm birth has not been fully elucidated.

### Sex differences

Sex-specific differences in responses to prenatal maternal stress and adverse birth outcomes have been reported in the literature (44–51). Female and male fetuses may be affected differently by maternal stress, leading to differences in observed adverse birth outcomes (45). For example, maternal stress may adversely affect female fetuses by inducing adaptations such as slow growth (intrauterine growth restriction) or alterations in the enzyme 11 β -hydroxysteroid dehydrogenase type 2 which regulates access of glucocorticoids to steroid receptors (52). In a more recent study looking at sex specific differences and adverse birth outcomes, male fetuses whose mothers experienced more prenatal stressors (negative life events) had a shorter gestational length and higher rates of preterm birth compared to female fetuses (46). The biological mechanisms for these sex-specific differences in birth outcomes in response to maternal stress have not been clarified but placental and fetal hormonal mechanisms have been implicated (49–54).

### Purpose of the study

Whereas, the impact of pregnancy stress on adverse birth outcomes has been studied in high income countries, this has not been the case in low to middle income countries and especially Sub- Saharan Africa where research in this field is very scarce. Our study attempted to address this gap through collection of prospective data from a population of pregnant women in rural Kenya, a low-to-middle income African country with a high prevalence of preterm birth (5). Our study aims were twofold. Firstly, we determined the relationship of maternal psychological stress and stress-related hormones during the 2nd trimester of pregnancy to the length of gestation. For our second aim, we examined the moderating roles of maternal obstetric risk and fetal sex in the relationship between these stress-related metrics and gestational length.

Based on previous research, we hypothesized that maternal physiological stress (as measured by cortisol and cortisone levels) may be a better predictor of gestational length compared to psychological stress (as measured by self-reported perceived stress). We also proposed that both maternal obstetric risk and fetal sex would have influential effects on the relationship between pregnancy stress (physiological and psychological) and gestational length. Our study is the first to examine these potential moderating relationships. Understanding these relationships may help to explain previous mixed results regarding the role of perceived stress and physiologic stress in early delivery and preterm birth. Improved knowledge about these moderators can ultimately help to better individualize care and target preventive interventions where they are most needed.

## Methods

### Participants

This cohort study was conducted at the antenatal clinics of rural Migori County in the Western Province of Kenya between April 2017 and February 2018. Pregnant women were recruited between 22 and 28 weeks of gestation as dated by the first day of their last menstrual period. Many women from rural and under-served areas do not access care until this time in gestation and it was important to enroll women from these groups. We limited the gestational span for recruitment to 6 weeks in order to standardize the period when we assessed our stress measures as much as possible across participants. Participants included women aged 18–45 years with a singleton pregnancy, able to speak and understand at least one of three languages (Dholuo- the most frequently spoken local language, Kiswahili-National language or English) and who planned to deliver at either the Migori County and Referral Hospital or St Joseph's Catholic Mission Hospital in Ombo. From the preliminary data we collected from both recruitment sites, 60–70 percent of women seen at the antenatal clinics delivered in the same facility.

Women were excluded if they had twins or a higher order gestation, their fetus was known to have structural or genetic anomalies, the pregnancy was achieved by artificial technologies, or women had one of the following conditions: placenta praevia or abruption, known uterine or cervical anomalies, chronic renal and heart disease, diabetes mellitus, hypertension, thyroid disease, adrenal disease and HIV infection or AIDS. Women who were using steroid medications and those that had used peroxides or chemicals to bleach their hair were also excluded.

### Procedures

#### Recruitment and initial data collection

Ethical approval was sought and granted by institutional research boards at The Aga Khan University Hospital in Nairobi, Kenya and the University of California in San Francisco, USA. Women who fulfilled the inclusion criteria and gave consent to participate in the study underwent an obstetric ultrasound to further ascertain their gestation at recruitment. They also completed Cohen's Perceived Stress Scale to measure their levels of self-reported psychological stress together with a clinical and sociodemographic questionnaire. The women then provided a hair sample for cortisol and cortisone assay.

#### Follow up data collection

Participants provided two mobile phone numbers to the research team (self and spouse or next of kin) and were also given mobile numbers of the research assistants (RA). Women were instructed to call the RAs in case of any pregnancy complications or admissions to hospital and, importantly, to call when admitted to hospital for labor and delivery or immediately after delivery before discharge from hospital. They were also followed up with a monthly phone call from the research assistants till delivery. Women were given a small financial compensation for their time in completing study procedures.

A unique identifier was placed on each participant's prenatal card and medical record for ease of identification. The principal investigator and RAs conducted several research sensitization meetings with staff at the antenatal clinics, labor and delivery suites, and radiology to enhance their understanding of study goals and facilitate data collection during recruitment and follow up to eventual delivery.

The RAs monitored the antenatal wards, labor and delivery suites and postpartum wards on a daily basis to identify gestational progress of study participants. RAs also tracked deliveries that occurred outside the designated study hospitals (including home deliveries) to minimize loss to follow up. After delivery and before discharge from hospital, RAs reviewed participants' medical records to document gestational length, identify their number of obstetric risks on the Obstetric Medical Risk Index (OMRI), and extract other clinical data for assessment of potential covariates and descriptive information.

### Measures

#### Sociodemographic and clinical questionnaire

This self-report tool provided descriptive information about the sample and about potential confounds that could influence testing of the aims. Data included pre-pregnancy and pregnancy related medical conditions and measures such as weight and body mass as well as demographic, economic, nutritional, environmental and socio-cultural factors. Scientific evidence, clinical knowledge, and expertise regarding the local context (8, 9) informed covariates that were collected and examined in analysis of the aims. Culture and linguistic idioms were considered in developing the questionnaire.

#### Obstetric medical risk index

A score on Lobel et al. OMRI (42, 43) is based upon information collected from the woman's medical record. Thirty-seven pregnancy-related health risks are given a binary score as being present or not for each woman. All risks that are present are summed to create a woman's total score for obstetric risk. Major domains in the measure include patient health history (e.g., hypertension), family history (e.g., diabetes), complications of past pregnancies (e.g., pre-eclampsia), gynecological and obstetric history (e.g. previous spontaneous abortion), current pregnancy conditions (e.g., vaginal bleeding, substance abuse), and unusual features of the pregnancy (e.g., polyhydramnios). Scores range from 0 to 37 with higher scores indicating greater obstetric risk. Content validity of the items was derived from meta-analysis of key risks of adverse birth outcomes and clinician consensus ratings. The measure has demonstrated excellent predictive validity in relation to adverse birth outcomes (42).

#### Perceived stress scale

Cohen's Perceived Stress Scale (55) has 10 items, each on a 5-point Likert scale, which assess individuals' feelings and thoughts about how unpredictable, uncontrollable and overloaded they find their lives. The PSS has been used worldwide as a tool to measure perceived stress and has been validated widely in different languages and cultural contexts. It also has reliable psychometric properties with Cronbach alphas of between 0.69–0.91. The internal consistency of the measure with our sample was α = 0.83. For this study, we translated the measure into Dholuo (most spoken language in Migori) and Kiswahili (Kenya national language) and pre-tested its feasibility with 10 pregnant women attending the antenatal clinic at the Migori County and Referral Hospital. Women completed a print version of the translated measure. They were then asked the questions verbally by the interviewer to determine congruence between their verbal and written responses. The interviewer reported a high level of congruence between the two responses. Women who participated in the feasibility assessment reported items were easy to understand and they had no difficulty grasping the overall meaning of the questions asked. As a result, the PSS was administered in the original translated form.

#### Hair cortisol and cortisone

Hair samples were used to assess glucocorticoid levels. In contrast to saliva or plasma specimens, hair cortisol and cortisone reflect longer term HPA axis regulation over the prior months. This provided a retrospective assessment of persistent rather than acute or transient stress (56, 57). Research assistants were trained to collect and store hair samples using a standardized procedure, including written and pictorial instructions (58). Each hair sample consisted of approximately 20–30 strands, 3–4 mm thick and 3 cm long cut close to the scalp. The end closest to the scalp was marked with tape and the specimen was stored in an aluminum foil placed in a ziplock bag. Prior research indicates that a 3 cm specimen represents the cumulative cortisol and cortisone secretion of the previous 3 months (58).

Both cortisol and cortisone (a down-stream metabolite of cortisol) were examined for a more robust assessment of glucocorticoid activity. Hair cortisol (HCC) and cortisone (HCNC) concentrations were assayed in the TB Hair Analysis Lab at UCSF *via* liquid chromatography-tandem mass spectrometry (LC-MS/MS). An Agilent LC 1260 (Agilent Technologies, Sta. Clara, CA, USA)-AB Sciex API 5500 triple quadrupole mass spectrometer (AB Sciex, Foster City CA, USA) system was used, equipped with a Synergi Polar-RP column (2.1 x 100 mm, 2.5 μm particle size). Hair samples were first washed thoroughly with 2.5 mL isopropyl alcohol before being prepared by pulverization using an Omni Bead Ruptor® tissue homogenizer. Internal standards (cortisol d4- at a final concentration of 0.01 ng/mg hair) were added to pulverized hair, and cortisol and cortisone were then extracted by methanol for 2 h at 37°C. Extraction was repeated twice by vortexing for 15 min with methanol at 37°C. Supernatants from each extraction were combined and evaporated. The extract was re-suspended in 2% methanol and cleaned by solid phase extraction using Waters Oasis HLB column (1 cc, 10 mg). The final SPE eluate was evaporated and the resulting extract was re-suspended in 100 μL 15% methanol solution with 0.1% formic acid and 2 mM ammonium acetate.

Two 25 uL aliquots of each sample extract were injected into the LC-MS/MS for analysis using a mobile phase system consisting of water with 0.1% formic acid and 2 mM ammonium acetate (mobile phase A, MPA) and methanol with 0.1% formic acid and 2 mM ammonium acetate (mobile phase b, MPB). Cortisol and cortisone were separated by gradient elution using the following scheme: 0–0.5 min, 15% MPB; 0.5–1.92 min, 15–100% MBP; 1.92–2.92 min, 100% MPB; 2.93–5.00 min, 15% MPB. Mass spectrometric detection with positive ionization by electrospray ionization (ESI) and mass scanning was done *via* multiple reaction monitoring (MRM) using the following transitions: cortisol- 363.2–121.0 m/z, 363.2–77.0 m/z; cortisone- 361.2–163.2 m/z, 361.2–121.2 m/z; and cortisol-d4, 367.3–121.0 m/z. Quantitation of each hormone was done by isotope dilution method using a ten-point calibration curve and cortisol-d4 as an internal standard. AB-Sciex Analyst 1.6 and Multi-Quaint 2.1 software packages (AB Sciex, Foster City, CA, USA) were used for data analysis. Two quality control (QC) samples (high and low) were run alongside the samples at each batch. Data obtained from the analysis were only reported after determining that the QC values are within 20% of the target values and replicate measurements have coefficients of variation within 20%. Cortisol levels for 3 participants were extreme outliers (very high values that were not compatible with accepted ranges) so they were excluded. Cortisone and cortisol are reported in pg/mg.

#### Preterm delivery

Participants who delivered before 37 completed weeks were defined as having a preterm delivery.

#### Gestational length

This was calculated in weeks as the difference between the first day of the last menstrual period and the date of delivery and used as a continuous measure. An obstetric ultrasound done on the day of the recruitment was also used to ascertain gestational length, especially in cases where the woman was uncertain about the first day of her last menstrual period. The ultrasound dating was used to calculate gestational length if there was a significant discrepancy between the date of the last menstrual period and the ultrasound dating.

### Data analysis

Histograms, scatter-plots, and stem and leaf plots were used to check the distribution, linearity and outliers of the key predictor and outcome variables. Multiple imputation was used to address missing values. Missing cases ranged from 2 of 130 for PSS-10 to 5 of 130 cases each for obstetric risk and gestational age. The pattern of missing data was examined first, with review of the data suggesting that they were missing at random. We specified key variables to be analyzed in the imputation model (stress, obstetric risk, age, gestational age, cortisol and cortisone) and performed 20 imputations. We then exported the pooled results into our dataset to account for variation across imputations. Cortisol and cortisone distributions were positively skewed and were log-transformed to achieve normal distributions. Pearson correlations and *t*-tests were computed to examine the relationships of covariates that might need inclusion to control for their confounding effects. Maternal age, education, body mass index, systolic and diastolic blood pressure, and exposure to violence were examined as covariates. The relationship between stress-related variables and gestational length, as well as the moderating role of obstetric risk, were tested with linear regression procedures. In the model, gestational length (the dependent variable) was regressed on perinatal obstetric risk, perceived stress, hair cortisol level, and hair cortisone level, with an interaction term included to examine the moderating role of obstetric risk. Three separate regression models were computed to examine the interaction of obstetric risk with each of the stress-related metrics (perceived stress, cortisol and cortisone). The moderating role of fetal sex was tested in a similar way. In our fetal sex models, gestational length (the dependent variable) was regressed on obstetric risk, perceived stress, hair cortisol level, hair cortisone level, and fetal sex, with an interaction term included to examine the moderating role of fetal sex. As with the models for obstetric risk, three separate regressions were computed to examine the interaction of fetal sex with each of the stress-related metrics. In each of these models, any covariates showing a significant relationship to the dependent, predictor or moderating variables were included. We evaluated all tests of significance with a two-sided alpha of 0.05. A Bonferroni correction was applied to address multiple tests for each set of models.

## Results

### Sample

One hundred thirty women participated in the study. They had a mean age of 25 years (ranging from 16 to 40 years). On average, women were experiencing moderate stress, with a mean score of 19 (SD = 4) for those who delivered at term and 20 (SD = 3) for those who delivered preterm out of a total possible score of 40. In addition, their score for obstetric risk was 1.38 on average, with women's scores ranging from 0 to 8. Mean gestational length at delivery was 39 weeks for those who delivered at term and 34 weeks for those who delivered preterm. A similar proportion of female babies (13%) and male babies (14%) were born preterm. Table 1 provides detailed data on participant characteristics.

**Table 1:** Participants characteristics.

***N** =* **130**	**Term** ***N** =* **116**	**Preterm** ***N** =* **14**	***P*** **-value**
**Maternal age**			
Years (mean ± SD)	25 (±5)	24 (±4)	0.51
**Gravidity *n* (%)**			
1	42 (36)	6 (46)	0.46
2–3	58 (50)	5 (32)	
>3	16 (14)	3 (22)	
**Parity *n* (%)**			
0	42 (36)	6 (46)	0.60
1–2	56 (48)	5 (32)	
>3	18 (16)	3 (22)	
**Mode of delivery *n* (%)**			
Vaginal delivery	108 (93)	13 (93)	0.65
Caesarean delivery	8 (7)	1 (7)	
**Gestational age at delivery**			
Weeks (mean ± SD)	39 (±1)	34 (±2)	0.69
**Birthweight**			
Grams (mean ± SD)	3293 (±714)	2623 (±721)	1.0
**Fetal sex *n* (%)**			
Female	71 (61)	6(43)	1.0
**Marital status**			
Single	12 (10)	2 (14)	0.83
Cohabitation	32 (28)	3 (21)	
Married	72 (62)	9 (65)	
**Occupation *n* (%)**			
Housewife	55 (47)	5 (36)	0.62
Self employed	24 (21)	4 (28)	
Formally employed	37 (32)	5 (36)	
**Highest education level *n* (%)**			
Primary	37 (32)	3 (21)	0.68
Secondary	48 (41)	6 (43)	
Tertiary	31 (27)	5 (36)	
**Income per month (net) Kenya shillings (kes) *n* (%)**			
Less than 5,000	66 (57)	8 (57)	1.0
5001–20,000	36 (31)	5 (36)	
>20,000	14 (12)	1 (7)	
**Predictor variables mean (±SD)**			
Perceived stress	19 (±4)	20 (±3)	0.40
Hair cortisol (log transformed) pg/ml	5.89 (±1.04)	7.63 (±0.94)	0.26
Hair cortisone (log transformed) pg/ml	8.23 (±0.80)	8.61 (±0.86)	0.40
Obstetric risk	1.30 (±1.45)	1.33 (±1.76)	0.33

### Preliminary associations of covariates to key study variables

Table 2 provides correlations of the covariates with gestational age, obstetric risk, fetal sex, cortisol and cortisone. Age was significantly associated with obstetric risk so we included it as a covariate in all analyses.

**Table 2:** Pearson correlation coefficients for the relationship of potential covariates to key study variables.

	**Gestational length**	**Stress**	**Cortisol**	**Cortisone**	**Obstetric risk**	**Fetal sex**
Maternal age	−0.05	0.01	−0.03	−0.05	0.18*	0.08
Maternal education	0.10	−0.04	−0.01	0.00	−0.11	0.02
Systolic blood pressure	−0.02	0.05	0.02	0.08	0.06	0.03
Diastolic blood pressure	−0.04	0.07	−0.06	−0.10	0.10	0.04
Body mass index	−0.04	−0.07	−0.05	−0.06	0.15	0.02
Exposure to violence	−0.06	0.13	0.05	−0.02	−0.01	−0.02

*p < 0.05.

### The relationship of psychological and physiological stress to gestational length

Women's perceived stress was not significantly related to gestational length. Similarly, neither hair cortisol nor cortisone levels of women were significant, independent predictors of gestational length for the sample as a whole (Table 3).

**Table 3:** Linear regression models for effects of maternal perceived stress, cortisol, cortisone, and the moderating effect of obstetric risk on gestational length.

**Variable**	**B**	**SE**	**Beta**	**95% CI**	**P value**
**Perceived stress model**					
Age	−0.002	0.00	−0.04	−0.010, 0.006	0.63
Obstetric risk	−0.145	0.31	−0.16	−0.764, 0.474	0.64
Perceived stress	−0.002	0.01	−0.04	−0.017, 0.013	0.77
Risk x stress interaction	0.006	0.01	1.33	−0.026, 0.037	0.71
**Cortisol model**					
Age	−0.006	0.00	−0.12	−0.015, 0.003	0.19
Obstetric risk	0.133	0.27	0.14	−0.408, 0.874	0.62
Cortisol (pg/ml)	−0.024	0.04	−0.07	−0.111, 0.063	0.58
Risk x cortisol interaction	0.103	0.11	0.28	−0.117, 0.323	0.35
**Cortisone model**					
Age	−0.005	0.00	−0.11	−0.014, 0.003	0.22
Obstetric risk	1.320	0.32	1.39	0.078, 1.571	0.03
Cortisone (pg/ml)	−0.107	0.10	−0.13	−0.312, 0.097	0.30
Risk x cortisone interaction	0.709	0.30	1.50	0.104, 1.313	0.02

### Obstetric risk as a moderator of the relationship between stress and gestational length

The greater the number of obstetric risks experienced by women, the shorter was their gestational length (Table 3). Three obstetric risks were associated with the largest differences between women who delivered earlier in gestation and those who delivered later. First, 23% of women delivering earlier had genitourinary tract infections during pregnancy in contrast to only 13% of women who delivered later. Another difference was for the prevalence of influenza, with 13.6% of women who delivered earlier having the flu during their pregnancy while only 6.7% of women who delivered later contracted the flu. Lastly, 9% of women who delivered early had experienced a stillbirth in a previous pregnancy in contrast to only 1.7% of women who delivered at term.

**Figure 1:**
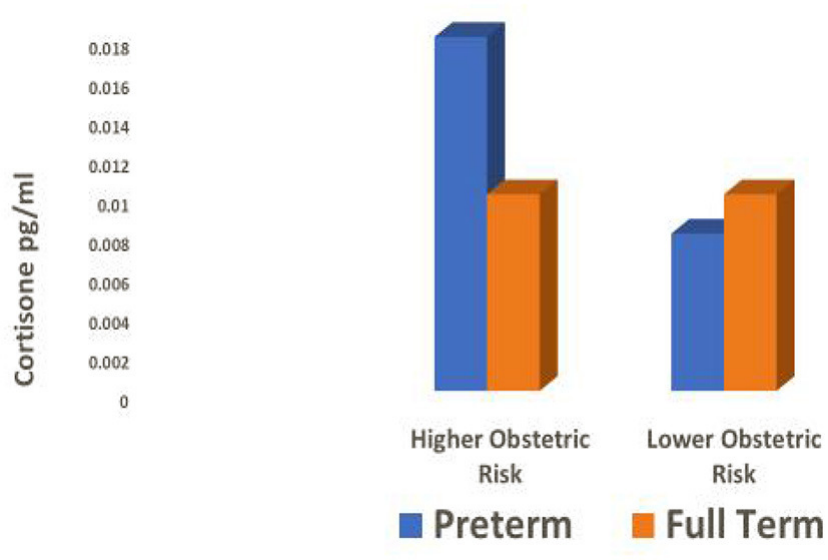
Hair cortisone levels during pregnancy by obstetric risk and birth outcome.

The effect of obstetric risk on gestational length was further explained by its significant interaction with cortisone (*B* = 0.709, *p* = 0.02). There was a significant difference in hair cortisone levels between women who delivered at 37 weeks gestation or less (x = 0.018 (0.01) pg/ml) and women who delivered after 37 weeks gestation (x = 0.010 (0.00) pg/ml; *t*(42) = 2.39, *p* = 0.02). However, this was true only among women who had 2 or more pregnancy-related risks on the Obstetric Medical Risk Index (OMRI). Cohens d for this difference was 0.98, a very large effect size. In contrast, there was no difference between cortisone levels of women who delivered earlier versus later and who had only one or no pregnancy-related risks on the OMRI (Cohen's d = 0.28). In comparing women who delivered preterm and full term, mean cortisone levels of term mothers were identical for higher and lower obstetric risk groups while cortisone levels of mothers who delivered prematurely were higher if they had higher obstetric risk than lower risk (Figure 1).

### Fetal/infant sex differences in the relationship between stress and gestational length

As shown in Table 4, fetal sex had no relationship to gestational length in any of the models. There were also no differences based on sex for obstetric risk, any stress-related variable, or for gestational length (Table 5).

**Table 4:** Linear regression models for effects of maternal perceived stress, cortisol, cortisone, and the moderating effect of fetal sex on gestational length.

**Variable**	**B**	**SE**	**Beta**	**95% CI**	**P-value**
**Perceived stress model**					
Age	−0.003	0.00	−0.07	−0.012, 0.005	0.43
Fetal sex	−0.099	0.18	−0.20	−0.465, 0.267	0.59
Perceived stress	−0.008	0.01	0.16	−0.038, 0.022	0.59
Sex x stress interaction	0.006	0.01	0.31	−0.012, 0.024	0.53
**Cortisol model**					
Age	−0.008	0.00	−0.16	−0.017, 0.001	0.08
Fetal sex	−0.104	0.15	−0.21	−0.408, 0.200	0.50
Cortisol (pg/ml)	0.095	0.09	0.29	−0.087, 0.277	0.30
Sex x cortisol interaction	−0.061	0.06	−0.38	−0.187, 0.065	0.34
**Cortisone model**					
Age	−0.008	0.00	−0.17	−0.017, 0.001	0.08
Fetal sex	−0.252	0.33	−0.51	−0.992, 0.418	0.46
Cortisone (pg/ml)	0.233	0.27	0.28	−0.302, 0.769	0.39
Sex x cortisone interaction	−0.138	0.18	−0.65	−0.459, 0.181	0.39

**Table 5:** Means of study variables for women who delivered female and male infants.

**Variable**	**Female**		**Male**	
	**M**	**SE**	**M**	**SE**
Gestational length	38.95	0.31	38.89	0.26
Obstetric risk	1.48	0.19	1.30	0.19
Perceived stress	19.66	0.50	19.82	0.65
Cortisol (pg/ml)	0.088	0.05	0.049	0.04
Cortisone (pg/ml)	0.010	0.00	0.010	0.00

## Discussion

### Key findings

Scarce research world-wide has examined the relationship of hormonal and psychological stress to gestational length or the moderating effects of obstetric risks and fetal sex on this relationship. In particular, nothing is known of these relationships among African women. We found that perceived stress, hair cortisol and cortisone were not significant independent predictors of gestational length among rural Kenyan women. However, the risk of shorter gestation increases with every additional obstetric risk incurred by a woman. This effect was further explained by the interaction between obstetric risk and hair cortisone. Hair cortisone levels of mothers who delivered earlier in gestation were significantly higher than for women who delivered later, but only among women who had high obstetric risk during pregnancy. Fetal sex had no relationship to gestational length nor any moderating effect on the relationship between any stress-related metric and gestational length.

### Obstetric risk and gestational length

Our findings regarding the importance of obstetric risks to gestational length are congruent with a growing body of research showing links between varied obstetric risks and preterm delivery (34, 35). The particular risks we observed among women who delivered earlier support previous research as well. Genitourinary tract infections have been associated with preterm birth in a number of recent studies (59), including in Rwanda (60) and Ethiopia (61). Similarly, there is strong support for the role of influenza (62–65) and a history of stillbirth (66, 67) in preterm delivery. All three obstetric risks may have a common mechanism underlying their effects. There is evidence that enhanced pro-inflammatory responses to infection are detrimental to pregnancy, with elevated levels of pro-inflammatory cytokines predicting preterm labor (68). In addition to the clear-cut role of inflammation in genitourinary and influenza infection, bacterial and viral infections have also been implicated in the etiology of stillbirths in both developing and industrialized countries (69). Networks of interacting cytokines help to maintain homoeostasis during pregnancy. Strong or dysregulated inflammatory responses that disturb these networks may trigger physiologic changes which precipitate early parturition through processes such as reduced fetoplacental blood flow or greater production of prostaglandins (69, 70). In light of this possibility, screening for infection early in gestation seems essential. Identifying and treating both genitourinary bacterial infections and viral infections associated with influenza may decrease the risk of preterm delivery (71).

### Elevated cortisone and shorter gestation among women with high obstetric risk

Cortisone (a metabolite of cortisol) has recently emerged as an important biological marker of interest in studies exploring maternal psychological distress during pregnancy. Research has shown evidence for cortisone as a potentially more salient marker of physiologic stress during pregnancy than cortisol (57, 58). Our finding in this research supports the importance of cortisone as a stress-related biomarker, indicating that higher levels of cortisone (but not cortisol) were present in the hair of women with greater obstetric risk who delivered earlier in gestation than among high-risk women who delivered later. During pregnancy, approximately 90% of cortisol is converted to inactive cortisone by the enzyme 11β-hydroxysteroid dehydrogenase type 2 [11βHSD2; (72, 73)]. This conversion is viewed as a mechanism through which the hypothalamic-pituitary-adrenal (HPA) axis (the body's stress regulation system) protects the fetus from adverse effects of excessive cortisol levels (72). Elevated glucocorticoids (both endogenous and exogenous) have been associated with preterm birth in previous research (74–77). It is likely that women with more obstetric risk, especially those with infectious processes that enhance inflammation, had higher glucocorticoid levels that put them at greater risk of preterm birth. A critical role of the HPA-axis is regulation of inflammation, including down-regulation of pro-inflammatory cytokine production (78). As we noted earlier, the major obstetric risks found among women in our sample who delivered earlier in gestation appeared to involve infectious processes that cause inflammation in the genitourinary and uteroplacental tracts. Elevated levels of cortisone may have reflected increased immunomodulatory activity exerted by the HPA axis to suppress inflammation.

Alternatively, elevated cortisone levels, as an indicator of physiological stress, may have placed women at greater risk for infectious processes that increased their vulnerability to shorter gestation. Accumulating evidence suggests that glucocorticoids such as cortisone can have permissive effects on the immune system under specific conditions (78, 79). Enhanced cortisone production in response to stressor-induced HPA-axis activation may increase susceptibility to infectious disease or augment its severity through suppression of immune activity that typically controls or eliminates pathogens (78, 79). Development of infection, in turn, could then increase vulnerability to early delivery or preterm birth (67).

### Psychological stress and shorter gestation

Our null findings regarding perceived stress support a growing body of research suggesting that physiological stress may be a greater risk factor for early delivery than psychological stress (68, 75). However, we used the Perceived Stress Scale, a global measure of general stress, to measure psychological stress. Some research indicates that pregnancy-specific stress (e.g., concerns about fetal health, impending childbirth, or changes in the body) may be a better predictor of adverse birth outcomes, including preterm birth (41). The effects of type of stress and its timing during pregnancy warrant attention in future research.

### Fetal sex as a moderator

As we noted in the introduction, some literature suggests that male and female fetuses respond differently to maternal stress with subsequent sex-specific differences in birth outcomes (44–48). It has been proposed that these differences may stem from interactions of the maternal and fetal HPA axes, the placenta, the autonomic nervous system, immune system, and other yet undetermined mechanisms (49–54). Still, conflicting results have been reported with varied studies suggesting that male fetuses or female fetuses may be more vulnerable to stress and yet others finding no sex differences (44, 45, 48, 49, 80, 81). Our study did not demonstrate any moderating effect of fetal sex on relationships between stress-related measures and gestational length.

### Limitations and strengths

Our study had several limitations. Both hair cortisone and cortisol together with perceived stress were only measured at one point in time during pregnancy (between 22 and 28 weeks of gestation). Serial measurements over all trimesters might have yielded different results as seen by the Hoffman et al. (31) study. Hair segmental analysis to examine shorter, more specific time periods during pregnancy was not done. Studies have shown that different hair segments may yield time-specific associations between prenatal stress and preterm birth. We used a global measure of psychological stress which had not undergone full psychometric testing for our population. Use of pregnancy-specific assessments in conjunction with general stress measures may be more fruitful in studying prenatal stress. We did not examine a wide swath of mental health covariates or other measures of biological stress, nor did we control for hair washing, seasonal variations or hair complexion.

The study had several strengths. We assayed both hair cortisone and cortisol. We studied the second trimester of pregnancy, a critical period for fetal development during which the fetus transitions to a state of possible viability and adaptation to extra-uterine life. Fetal organ systems are undergoing maturation to support extra-uterine life, making this time period especially vulnerable to changes in the internal or external milieu such as maternal stress. This is one of very few studies worldwide, and a first of its kind in Sub Saharan Africa, to explore the associations of maternal stress, hair cortisol and cortisone to adverse birth outcomes.

## Conclusions

In this study, we found that higher levels of second trimester maternal cortisone were associated with shorter gestation among women with higher obstetric risk. Hair cortisone levels may be an important stress-related biomarker in the prediction of preterm birth. In contrast, hair cortisol and maternal perceived stress were not associated with earlier delivery. There were no moderating effects of fetal sex in the relationship between maternal stress and gestational length.

Future studies should incorporate both hair cortisone and cortisol assays as biological measures of stress. These assays can be taken at multiple time points in pregnancy and incorporate segmental hair analysis to elicit more nuanced effects of potential HPA axis dysregulation on birth outcomes. Pregnancy-specific psychological measures administered at different time points in pregnancy (especially those that have undergone full psychometric testing for the intended study populations) are recommended for future studies. Sex-specific differences in adverse birth outcomes warrant further study to understand their underlying biological mechanisms, including epigenetic and gene expression studies. Such studies might offer further explanations regarding sex-specific fetal effects of maternal stress. Lastly, our research indicates that the moderating effect of obstetric risk is essential to consider in examining the relationship between stress-related measures and shorter gestation. In addition, assessment of risk-related inflammatory markers such as cytokines and TNF-*a* will be important in order to better understand the potential effects of the relationship between the immune system and the HPA-axis on increased vulnerability to preterm birth.

## Data availability statement

The raw data supporting the conclusions of this article will be made available by the authors, without undue reservation.

## Ethics statement

The studies involving human participants were reviewed and approved by Institutional Research Boards of the University of California San Francisco and Aga Khan University Hospital Nairobi Kenya. The patients/participants provided their written informed consent to participate in this study.

## Author contributions

JM wrote the original research concept, research proposal, study implementation, data analysis and the original manuscript preparation. CC reviewed the research proposal, supported research implementation and reviewed and edited the manuscript before publication. MK reviewed the research proposal and reviewed and edited the manuscript before publication. RG conducted the analysis of hair cortisol and cortisone and was involved in manuscript revisions before publication. AW was involved in research proposal development, study implementation and manuscript revisions before publication. DA worked with RG to process the hair samples to conduct and report on hair cortisol and cortisone analysis. NS was involved in the original research concept preparation, research proposal reviews, implementation meetings and manuscript review before publication. MT was involved in research implementation meetings and manuscript revision before publication. SW was the primary academic and research mentor for JM during his fellowship studies, contributed to the original research concept, development of the research proposal, study implementation, data analysis and writing of the manuscript. All authors contributed to the article and approved the submitted version.

## Funding

The study was supported by research funds from Bill and Melinda Gates Foundation grant number OPP1107312 and a gift from Marc and Lynne Benioff through the Preterm Birth Initiative Fellowship Program administered by the University of California in San Francisco. JM was in the inaugural cohort of fellows in the PTBi Fellowship Program and was the primary recipient of the research funds. SW was JM's research and academic mentor and a Co-Principal Investigator in this study.

## Conflict of interest

The authors declare that the research was conducted in the absence of any commercial or financial relationships that could be construed as a potential conflict of interest.

## Publisher's note

All claims expressed in this article are solely those of the authors and do not necessarily represent those of their affiliated organizations, or those of the publisher, the editors and the reviewers. Any product that may be evaluated in this article, or claim that may be made by its manufacturer, is not guaranteed or endorsed by the publisher.
