# Supplementary material for: Obstetric risk in pregnancy interacts with hair cortisone levels to reduce gestational length
Source: Front Glob Womens Health. 2022 Jul 22;3:878538. doi: 10.3389/fgwh.2022.878538 (PMC9354598; doi:10.3389/fgwh.2022.878538)
Supplement: Supplementary file 1 [file Data_Sheet_1.PDF]

ID ________

Obstetric Medical Risk Index

Use 0 if not present, 1 if present

Unusual features of pregnancy

1. ______diabetes (current or past)
2. ______heart disease (current or past)
3. ______polyhydramnios or oligohydramnios
4. ______communicable disease
5. ______Rh negative

Gynecological and obstetric history

6. ______5 or more past deliveries
7. ______previous fetal demise
8. ______previous stillbirth
9. ______previous preterm delivery
10. ______previous 2nd trimester spontaneous abortion
11. ______3 or more previous 1st trimester spontaneous abortions
12. ______2 or more therapeutic abortions

Complications of past pregnancies

13. ______hemorrhage
14. ______placenta previa or accreta
15. ______pregnancy induced hypertension
16. ______pre-eclampsia (toxemia)
17. ______gestational diabetes

Family history

18. ______diabetes

Patient history (can be identified in current pregnancy as long as not counted twice)

19. ______hypertension
20. ______pulmonary disease (or current asthma)
21. ______genito-urinary infections

Weiss, 2016_sandra.weiss@ucsf.edu

22. ______renal disease
23. ______epilepsy
24. ______uterine surgery
25. ______uterine or cervical abnormality
26. ______herpes

Current pregnancy complaints

27. ______anemia
28. ______flu syndrome
29. ______genito-urinary infections
30. ______incompetent cervix
31. ______alcohol abuse
32. ______substance abuse
33. ______placenta previa or accreta
34. ______postmaturity
35. ______threatened abortion
36. ______tobacco use
37. ______second or third trimester bleeding

______TOTAL Obstetric Risk

Adapted from Lobel et al 2000, 2008

1. Lobel M, Cannella D, Graham J, DeVincent C, Schneider J, Meyer B. (2008). Pregnancy-specific stress, prenatal health behaviors, and birth outcomes. *Health Psychology*, 27, 604–615. doi:10.1037/a0013242

2. Lobel M, DeVincent CJ, Kaminer, A., & Meyer, B.A. (2000). The impact of prenatal maternal stress and optimistic disposition on birth outcomes in medically high-risk women. *Health Psychology*, 19, 544–553. doi:10.1037/0278-6133.19.6.544
